# Progress in Surgery

**Published:** 1899-02-25

**Authors:** 


### PROGRESS IN SURGERY.

### RHINOLOGY.

(*Continued from page* 248.)

The recurrence of adenoids formed a topic in a recent discussion at the American Laryngological Association, being introduced by Ames Bliss.[17] He pointed out that it is not wholly correct to regard the pharyngeal tonsil as merely the upper segment of the lymphoid ring, as the pharyngeal tonsil in its development is distinct from that of the fauces, and is more or less intimately connected with the development of the pituitary body. Indeed, it is quite probable that this relationship in growth between the nasopharyngeal adenoid tissue and the base of the cerebrum immediately above it may be of much clinical importance in the development of pathological states within these areas. As the elongated diverticulum enlarges to form the developed pituitary body, within what will be, when completed, the sella turcica, the narrowing pedicle formed by its development, and connecting it with the pharyngeal cavity, is supposed to be obliterated; yet Lanzert has shown that in ten children out of one hundred examined a trace of this passage remained at birth, called by him the cranio-pharyngeus. It is quite probable that a larger number of examinations would show a corresponding percentage of cases in which this passage remains open; while the cases may not be very rare in which traces of it may persist in childhood or adult life. The much discussed bursa pharyngea may be a phase of such persistence of this passage. We know that as the hypophysis cerebri develops, its anterior portion becomes highly vascularised, and it is quite probable that numerous blood channels from this area lead to the adenoid tissue of the pharyngeal vault and to the circulation at the base of the cerebrum. Dr. Harrison Allen, in his "Clinical Studies of the Skull," has demonstrated from a large number of crania of all races that great variation in structure exists in the region of the vault at the articulation of the vomer with the vaginal process of the sphenoid and sphenoidal processes of the palate bone, variations characterised by failure in complete union of the parts named, the open areas between showing evidence of having been filled during life with vascular tissue. Allen terms these openings and passages *canales basis vomeris*. In many instances the wings of the vomer, he states, are quite free from the sphenoidal and vaginal processes, and are not in close articulation with the body of the sphenoid, numerous free openings leading in between the articulating surfaces. Bliss goes on to ask, "Is it not possible, considering the close relation during development between the future pharyngeal vault and the hypophysis cerebri, that in the living subject such open areas may not only contain blood vessels connecting these areas, but even extensions upward of the adenoid tissue in the nasopharynx?" Now Bliss has noted (1) that in his cases of apparent recurrence the whole general structure of the nasofaucial area was either infantile or else exhibited an abnormal oblique basilar process which appeared to articulate with the sphenoid considerably above the vomero-sphenoidal articulation, the recess being occupied by a mass of adenoid tissue; (2) the excessive vascularity of the hypertrophied lymphoid tissue; (3) in a few instances fine friable spicules of bone about the articulation of the vomerine wings and sphenoid in the roof of that area which Harrison Allen termed "the posterula"; (4) a strumous appearance—an apparent failure of those processes which tend to complete development. He thinks that "recurrence" may often be attributed to a failure to remove completely the whole mass of diseased tissue in the regions referred to, and he advocates complete removal of the adenoid tissue in all cases, especially of those portions which lie well forward about the vomero-sphenoidal articulation, a roof of the "posterula," and of those parts which lie in the lateral regions of the nasopharynx, above the upper border of the Eustachian openings and in Rosenmüller's fossæ. Bryson Delavan,[18] in a simultaneous communication, remarked that it is said by some that a partial removal of an adenoid growth will cause the remnant to atrophy, thus making complete removal unnecessary, adding that no proposition could be more fallacious. Granting that an actual disappearance of the remaining tissue may now and then take place, such cases must in reality be unusual. The tissue left behind is unhealthy tissue, and may become atrophic, and cause lifelong annoyance to the patient. On the other hand, during and before early adult life, as much tissue as has been left behind at the time of the operation is likely to remain, and the chances are almost as much in favour of its increasing as the contrary. Other things being equal, the earlier the age at which the growth has been imperfectly removed the greater the tendency to recur. He reminds us that both Wilhelm Meyer and Franklin Hooper strenuously insisted upon thorough work in removal. Of course, from the nature and distribution of the lymphoid tissue of the pharynx, it is practically impossible to literally remove all this tissue. Again, in very young children, the danger of inflicting injury upon the surrounding parts may deter us from operating as radically as would be proper in an older patient. Nevertheless the successful treatment of these cases demands the greatest possible thoroughness. Delavan emphasises the obviously painful character of all ordinary methods of removing adenoids, and therefore insists on the inhumanity of removing them without anæsthesia, and that to be efficient general anæsthesia is essential. The danger of separated lymphoid masses falling into the larynx has been alluded to by several writers, says Delavan, and, while Gottstein ("Heyman's Handbuch") considers it unworthy of serious thought, it is by no means an impossible risk. Again, serious injury has been inflicted upon the posterior edge of the nasal sæptum, the turbinated bodies, and the lateral walls of the pharynx in the neighbourhood of the Eustachian orifices, dangers likely to arise in operating upon a struggling child. He does not believe in the efficiency of the Gottstein knife, for he has often found that, in the hands of other operators, it has simply cut through the lower part of the growth, or has removed a certain amount from one side, leaving the other side full, or has fairly cleared the vault, leaving abundant deposits upon the posterior and lateral pharyngeal walls. He remarks that it must be seldom that the shape of the instrument is perfectly conformed to the pharynx; but in the blunt forceps, modified to suit the size of the pharynx and the location of the growth, we have an instrument by which the lymphoid masses can be seized, and, by proper manipulation, torn away, much larger fragments than the part caught in the grasp of the instrument usually being separated at each attempt. In skilled hands the forceps can be turned in all necessary directions, while the loss of blood is less than is common with sharp instruments. The snare, in its present stage of development, need not engage our attention. He urges greater caution than is usually employed in the after-care of the patient, and he should be put to bed for at least twenty-four hours. Bryson Delavan believes that the operation for the removal of adenoids is not the simple and easy thing that many suppose it to be, but, on the other hand, its successful performance requires considerable knowledge, experience, and skill.

Clarence Rice[19] remarked that one reason why we do not always get good results at once after operation is on account of the narrowing of the width of the superior maxillary bone, which by its upward pressure causes deflection of the nasal septum. Its abnormal relation to the inferior maxilla makes it difficult for the child to keep the mouth closed even though all post-nasal adenoid has been removed.

[17] New York Med. Jour., Oct. 29, 1898. [18] Ibid. [19] Ibid.

